# Robotics Dexterous Grasping: The Methods Based on Point Cloud and Deep Learning

**DOI:** 10.3389/fnbot.2021.658280

**Published:** 2021-06-09

**Authors:** Haonan Duan, Peng Wang, Yayu Huang, Guangyun Xu, Wei Wei, Xiaofei Shen

**Affiliations:** ^1^The State Key Laboratory for Management and Control of Complex Systems, Institute of Automation, Chinese Academy of Sciences, Beijing, China; ^2^Department of Information Science, School of Computing and Information, University of Pittsburgh, Pittsburgh, PA, United States; ^3^School of Artificial Intelligence, University of Chinese Academy of Sciences, Beijing, China; ^4^Center for Excellence in Brain Science and Intelligence Technology, Chinese Academy of Sciences, Shanghai, China

**Keywords:** robotics, dexterous grasping, point cloud, deep learning, review

## Abstract

Dexterous manipulation, especially dexterous grasping, is a primitive and crucial ability of robots that allows the implementation of performing human-like behaviors. Deploying the ability on robots enables them to assist and substitute human to accomplish more complex tasks in daily life and industrial production. A comprehensive review of the methods based on point cloud and deep learning for robotics dexterous grasping from three perspectives is given in this paper. As a new category schemes of the mainstream methods, the proposed generation-evaluation framework is the core concept of the classification. The other two classifications based on learning modes and applications are also briefly described afterwards. This review aims to afford a guideline for robotics dexterous grasping researchers and developers.

## Introduction

In the last decades, there has been an enormous proliferation in robotic community, both at in terms of research and attracting boundless varieties of imagination of general public, due to its diverse possibilities. The vast majority of robots in operation today consist of 6 degree of freedom (6-DOF) which are either rotary (articulated) or sliding (prismatic), with a simple end effector for interacting with the workpieces (Murray et al., 1994). Robot manipulation means it can use and control different objects according to certain specifications and essentials through the end effector to achieve the effect of making the best use of playing the role of object itself (Okamura et al., 2000; Saut et al., 2007). Grasping, as one of the most primitive manipulations, almost all high-level operations and complex tasks that people expect robots to complete are inseparable from the assistance of it. With the in-depth development of robotics, researchers begin to facilitate the transition from simple or even crude grasping of robots with less discrimination of objects to object-oriented dexterous grasping. Unlike simple grasping, dexterous grasping is able to determine which posture to be employed to grasp where of the object to ensure a higher grasping success rate (Ciocarlie et al., 2007; Prattichizzo et al., 2008; Ciocarlie and Allen, 2009).

**Figure 1:**
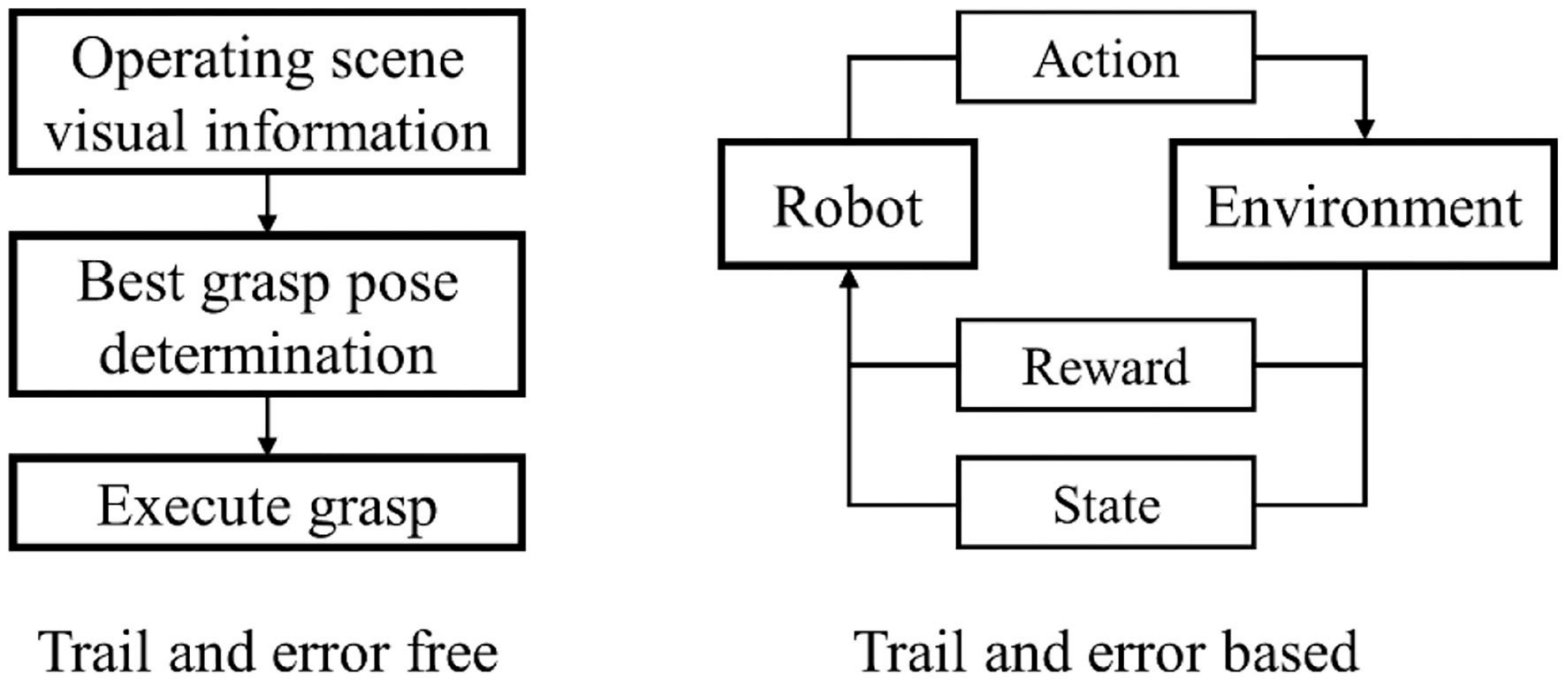
Recent dexterous grasp pipeline.

The research on dexterous manipulation and grasping can be traced back to the 1980s. In the era when deep learning methods were not yet established, researchers came up with ideas for dexterous manipulations from the perspective of physics and geometry. Through the kinematic modeling of robots, plenty of research results that attracted widespread attention at the time were born (Moreno et al., 2011; Fischinger et al., 2015; Chen et al., 2016; Zhou Z. et al., 2018; Zito et al., 2019; Monica and Aleotti, 2020). However, robot grasping algorithms based on physics and geometry presuppose many assumptions, making these methods hard to generalize. With the substantial increase in computing power from hardware, the artificial intelligence surge represented by deep learning methods has quickly penetrated into various research fields. Free from the limitation of manually extracting features, the grasping algorithms based on deep learning have achieved insurmountable effects in all aspects by traditional approaches, taking the robot's intelligence to a higher level. Specifically, with RGB images or depth images as input, robotic grasping based on convolutional neural network (CNN) which is a dominant deep learning framework in the field of computer vision, has obtained high grasping success rates in many tasks (Lenz et al., 2015; Varley et al., 2015; Johns et al., 2016; Finn and Levine, 2017; James et al., 2017; Kumra and Kanan, 2017; Zhang et al., 2017; Dyrstad et al., 2018; Levine et al., 2018; Schmidt et al., 2018; Schwarz et al., 2018). As shown in Figure 1, nowadays, based on visual information, robot dexterous grasp learning can be roughly divided into two categories based on whether the learning process is based on trial and error. Dexterous grasping learning that is not based on trial and error will determine the best grasp posture based on the visual information of the scene, and then execute it. On the contrary, the dexterous grasping learning based on trial and error is to accumulate the experience of grasping from failure through the interaction between the robot and the environment to improve grasping dexterity.

As a commonly used 3D visual data form, point cloud can be generated by 3D laser scanner (LIDAR), depth sensors or RGB-D images (Liu W. et al., 2019; Guo Y. et al., 2020; Lu and Shi, 2020). Compared with RGB or depth images, 3D point clouds can store more spatial information as their higher dimensions. With the improvement of point cloud processing methods (Fischler and Bolles, 1981; Rusu et al., 2010; Rusu and Cousins, 2011; Aldoma et al., 2012; Chen et al., 2016) and the introduction of CNN based on point cloud as input (Wu et al., 2015; Qi et al., 2017a,b), point clouds have become increasingly common for those tasks based on visual perception. Meanwhile, as more and more contributions on datasets of grasping based on point cloud (Goldfeder et al., 2009; Calli et al., 2015a,b, 2017; Kappler et al., 2015; Mahler et al., 2016, 2017; Depierre et al., 2018; Bauza et al., 2019; Bottarel et al., 2020; Fang H.-S. et al., 2020), robotic dexterous grasping based on point cloud and deep learning set off a tremendous wave of research in the field of robotics.

Based on the current work of robot dexterous grasping combining with point cloud and deep learning, this paper summarizes relevant important work from 2015 to present. As the earliest state-of-the-art work in related research, grasp pose detection (GPD) (Pas and Platt, 2015; Gualtieri et al., 2016; Pas et al., 2017) samples various grasp poses (candidate generation) in the point cloud and employ a deep learning method to assess these grasps (candidate evaluation) to obtain the optimal grasp pose. Inspired by this efficient pipeline, most subsequent works followed this framework to ameliorate generation or evaluation stages. From this perspective, this survey proposes a more generalized framework and summarizes relevant work as one or both of these two stages, that is, in which step the work contributes more. The articles reviewed in this paper are all based on deep learning framework. If deep learning strategy is not utilized in the generation stage, it will appear in evaluation stage, and vice versa. Remaining uncategorized models are provided in a separate section afterwards. The taxonomies from different perspectives of learning modes and applications are also briefly described. This paper aims to provide valuable insights and inspirations through the proposed taxonomy.

The remainder of the paper is organized as follows. Section Proposed Taxonomy presents related surveys and proposed category scheme. Section Grasping Candidate Generation and Grasp Candidate Evaluation are about the methodologies of grasp candidate generation and evaluation respectively. The uncategorized papers out of proposed framework are described in section End-to-End and Others. Section Learning Modes summarizes the methods from learning modes. In section Applications, it mainly introduces the applications of related approaches from perspectives of end effectors and operating scenarios. Section Challenges and Future Directions provides the challenges and future direction of this field. Section Conclusion is the conclusion of the paper.

## Proposed Taxonomy

As listed in Table 1, there are already numerous surveys in the field of robotics learning. Some surveys elaborate the perception techniques of robotics, and some others introduce approaches of robot manipulation. Ruiz-del-Solar et al. (2018) and Du et al. (2020) pay attention to the vision methods for robot manipulations. With the exception of visual perception approaches, Luo et al. (2017) and Wang C. et al. (2020) exhibit there are many other perception methods can help improve robot performance. Caldera et al. (2018), Kroemer et al. (2019), Li and Qiao (2019), and Kleeberger et al. (2020) focus on the overview of robot manipulation methods based on deep learning. Mohammed et al. (2020) and Zhao W. et al. (2020) introduce the techniques in robot learning on the basis of reinforcement learning. Billard and Kragic (2019) describes the trends and challenges in robot manipulation.

**Table 1:** The related surveys and corresponding topics.

**References**	**Review topic**	**Journals**
Du et al., 2020	Vision methods facilitate grasp estimation	Artificial intelligence review
Ruiz-del-Solar et al., 2018	Deep learning methods for robot vision	arXiv
Luo et al., 2017	Robotic tactile perception	Mechatronics
Wang C. et al., 2020	Feature sensing and robotic grasping	Sensors
Caldera et al., 2018	Deep learning methods in grasp detection	Multimodal technologies and interaction
Kroemer et al., 2019	Learning-based methods in robot manipulation	arXiv
Kleeberger et al., 2020	Learning-based robotic grasping	Current robotics reports
Li and Qiao, 2019	Robotic grasping and assembly tasks	IEEE Transactions on mechatronics
Mohammed et al., 2020	Deep reinforcement learning-based grasping	IEEE Access
Zhao W. et al., 2020	Sim-to-real problems of reinforcement learning	arXiv
Billard and Kragic, 2019	Trends and challenges in robot manipulation	Science

Unlike the works mentioned above, this paper focuses on the methods of robotic dexterous grasping based on point cloud and deep learning. Compared with previous related reviews, this paper narrows the reviewed works scope through the limitation of inputs and methods, aiming to provide a more detailed description in a specific direction. For the robotic grasping algorithms, the following four classifications have appeared in past researches but not entirely suitable for the topic in this paper. (1) Analytic-based and empirical-based: because this survey pays attention to the use of deep learning, traditional analytic methods are not within the scope of the review, so this classification is not applicable. (2) Task-agnostic and task-specific: this will complicate the classification of reviewed approaches mainly focus on grasping in this paper. Some task-specific papers will outperform under some specific circumstances, but don't have generalization ability, which cannot support a category. (3) Vision-based and vision-free: since this paper is specifically aimed at point cloud-based robotic grasping, most of the networks used are CNN-based, even numerous methods don't explicitly perform object recognition, segmentation, or pose estimation. In other word, the reviewed articles in this paper can be said use visual information explicitly or implicitly. Therefore, this classification is not appropriate. (4) Learning-based and learning-free: this classification is similar to (1). If each stage in the proposed pipeline is not based on learning, this method will not be taken into account in this survey.

**Figure 2:**
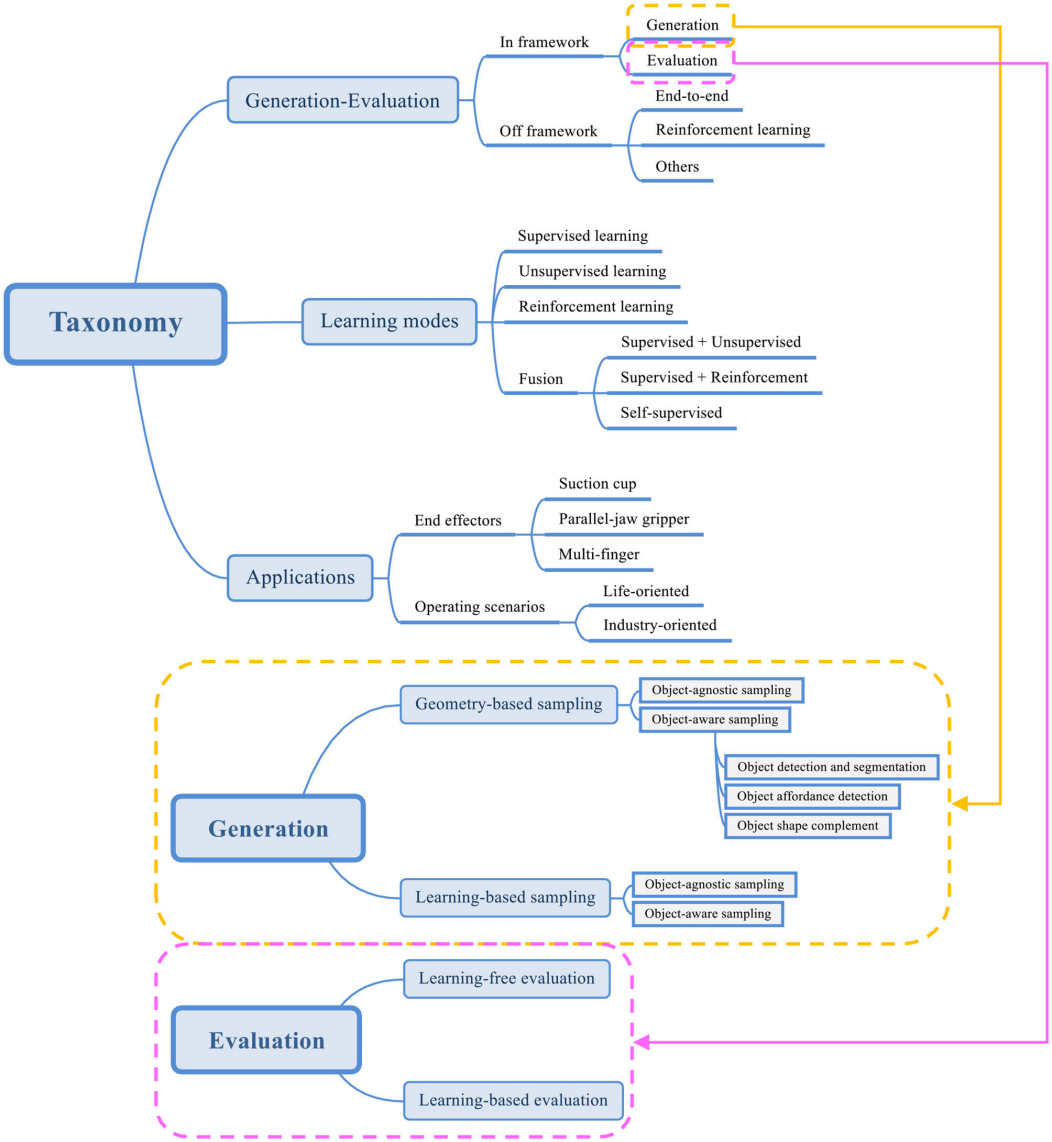
Robotics dexterous grasping methods based on point cloud and deep learning.

In order to elaborate the subject more comprehensively, instead of adopting the existing classification methods, this survey classifies related work from three perspectives: generation-evaluation, learning modes, and applications as shown in Figure 2. The proposed generation-evaluation framework is the core part of this paper. In the stage of grasp candidate generation, the methods can be divided into geometry-based sampling and learning-based sampling. In geometry-based sampling, methods are categorized into object-agnostic sampling and object-aware sampling. In object-aware sampling, object detection and segmentation are the most basic methods, object affordance detection and object shape complement are the further improvements. The learning-based sampling can also be divided into object-agnostic sampling and object-aware sampling. Unlike the methods in geometry-based object-aware sampling, there is no more classifications in the branch of learning-based object-aware sampling. In the grasp candidate evaluation stage, methods are split into learning-free and learning-based. This paper will use two separate sections to elaborate the methods of grasp candidate generation and evaluation. The following section will introduce some work that cannot be classified as this framework. They fall into end-to-end learning-based, reinforcement learning-based and others. Two remaining classifications will be briefly explained afterwards.

## Grasping Candidate Generation

**Figure 3:**
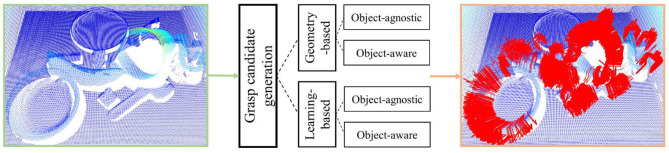
The general pipeline of grasp candidate generation.

Grasp candidate generation, also called grasp pose sampling, refers to randomly sampling the parameters (the approaching direction of the end effector, opening size, joint angle, etc.) of the end effector on the target object within a specific range to obtain a large number of possible grasp gestures as shown in Figure 3. In order to ensure the optimal or suboptimal grasp posture can be found, the final executed grasp pose is commonly not directly calculated, but a large number of grasp candidates are sampled on numerous points in point cloud with random grasp configurations, so as to promise the reliability of results on the basis of quantitative advantages (Eppner et al., 2019). In general, sampling can be roughly divided into geometry-based sampling and learning-based sampling. In these two categories, approaches can be deeply split into object-agnostic sampling and object-aware sampling. The papers mainly contribute to generation stage are provided in this section.

### Geometry-Based Sampling

In the early work, researchers try to apply random sampling to gain grasp candidates, the feasibility of sampling method to generate reliable and reasonable grasping candidates is discovered and verified afterwards (Boularias et al., 2014, 2015). Based on this, with superior interpretability and intuitiveness, the geometry-based grasp pose sampling came into being (Pas and Platt, 2015; Wang and Ling, 2016; Pas et al., 2017). These methods add geometric restrictions to the sampling process, such as the grasp position as close as possible to the center of gravity of the object, or the size of the object in the grasp approaching direction cannot exceed the maximum width of the end effector, so that make the random generation more reasonable. The idea behind this method is to introduce manually calculated features into the hypothesis space of grasping candidates, improves the possibility of finding the optimal or suboptimal grasp posture. Thanks to its generation process is based on the modeling of the grasping model from the real world (Murray et al., 1994), although it is an earlier branch of the generation method, geometry-based sampling is still being adopted by a lot of work.

In general, the current work using geometry-based grasping pose sampling can be divided into two categories based on whether it has the specific information of the object to be grasped, namely, object-agnostic sampling and object-aware sampling. Since the point cloud describes the spatial information of the scene, for the object-agnostic sampling methods, even if there is no specific information of the object, they can also obtain suitable sampling points for generating grasping poses by the spatial information. For the methods of object-aware sampling, they will first extract the specific information of the object through the methods of computer vision, relying on pure point cloud or combining the information of RGB images and depth images, and then perform sampling on these higher-level information.

#### Object-Agnostic Sampling

Sampling based on physical and geometric constraints could have an impressive performance on robotic grasping, especially in the two parallel-jaw grippers community since mathematical models of robotic grasping was well-defined in the past few years (Murray et al., 1994; Okamura et al., 2000; Prattichizzo et al., 2008; Prattichizzo and Trinkle, 2016). Researchers propose numerous sampling methods by combining the established mathematical theories and task-specific conditions. Since the specific information of the object is not used to help the sampling, this kind of object-agnostic grasping pose sampling must rely on complete grasping mathematical modeling and a large number of physical and geometric constraints in specific tasks to achieve grasping candidate generation. For example, only points higher than the operating plane calculated through the point cloud may be belonging to the object, and then it is possible to result in some feasible grasping poses by sampling at these points. Although the calculation steps are relatively cumbersome and plenty of limitations are based on experience and modeling, in the age when deep learning has not yet been developed, there are still many attempts at this sampling method.

Pas and Platt (2015) and Wang and Ling (2016) define hypotheses contain position and orientation information of the graspable point, as well as its neighborhood points calculated by Taubin quadric fitting (Taubin, 1991; Pas and Platt, 2013). To make the sampling process more flexible, GPD (Pas et al., 2017) selects N points uniformly at random from the region of interest (ROI) of point cloud and then perform grid search on picked points to extract grasp configurations that satisfy the geometric reasoning. Several research works obtain impressive experimental results by adopting this idea (Mahler and Goldberg, 2017; Mahler et al., 2017, 2018; Viereck et al., 2017; Liang et al., 2019). Lou et al. (2020) takes a further step by randomly associate a pose with each sampled point. There are also some methods not using uniformly sampling strategy. Gualtieri et al. (2016) and Kiatos and Malassiotis (2019) calculate a surface normal and an axis of major principal curvature of the object surface in the neighborhood of the sampled point. Grasp candidates are then generated at regular orientations orthogonal to the curvature axis. Zhou et al. (2019) samples the grasping candidates based on the depth descriptor Depth Likelihood Volume (Zhou Z. et al., 2018).

Since the specific information of the target object is not required, the advantages of the object-agnostic sampling method in some aspects are very obvious. These methods do not require object segmentation, thereby avoiding errors caused in the segmentation stage that will affect the accuracy of subsequent grasp candidate generation. At the same time, these methods do not need to know the identity, class and shape of the target object, which also makes it possible to apply the grasp poses sampling algorithms on unknown objects in an open environment. Finally, these methods do not try to register a CAD model of the object to the point cloud, which could be very challenging. However, the strategy of not combining the specific information of the object will also bring inevitable shortcomings to these methods. First of all, these methods have high requirements for input. The quality of the point cloud will immediately affect the reliability of the candidate sampling results. This also indirectly causes the result of sampling on the point cloud extracted by the monocular camera to be much worse than that of the multi-view camera. Since the algorithm does not segment the objects, it can detect “grasps” that treat multiple objects as a single atomic object. This type of error is unusual with small-aperture hands, but one would expect it to become a more significant problem when the hand is physically capable of grasping larger objects. This also makes it reasonable for the algorithm's sampling results to perform poorly in cluttered scenes and collision detection. Moreover, these methods are going to become infeasible for the multi-finger robots. Almost all of techniques mentioned above focus on robots with a vacuum cup or parallel-jaw gripper for their end-effectors. Multi-finger end-effectors will introduce a rich-contact points problem which is more difficult for these methods to handle.

#### Object-Aware Sampling

**Figure 4:**
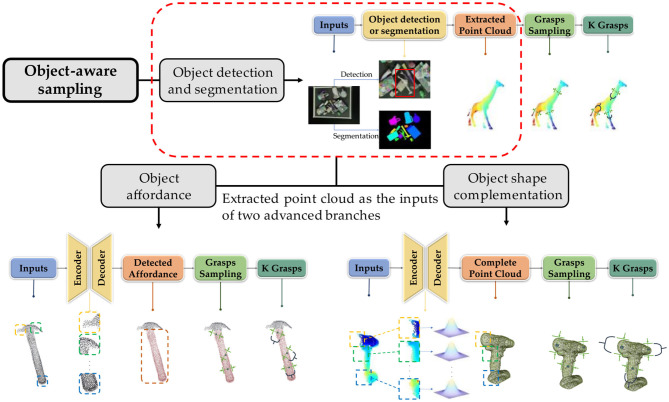
Entire pipelines of three classifications in object-aware sampling. Object detection and segmentation is the most basic method. Inputs are commonly RGB images, which are detected or segmented by networks to extract the object point clouds. Extracted point cloud can either be utilized to sample the grasp candidates immediately or fed into object affordance or shape complementation methods. Object affordance methods take extracted point cloud as inputs to obtain the affordance of object to reduce the sampling search space. On the contrary, object shape complementation aims to acquire the entire object point cloud to improve the grasp candidate generation confidence (The hammer point cloud is from YCB datasets).

Even object-agnostic sampling is able to find numerous grasp candidates, it doesn't use the complete information in point cloud. The drawback causes the low sampling accuracy and time-consuming sampling process. To avoid these defects, researchers propose object-aware sampling which aims to combine the specific information of the object to enhance the reasonability of search space in point cloud (Boularias et al., 2015; Zapata-Impata et al., 2017; Lopes et al., 2018). The search space refers to the points that need to be considered in the point cloud space for the grasping pose sampling algorithm. Since the object-agnostic sampling does not have the pose information of the object, those methods have to calculate all the points in the scene point cloud one by one. This is undoubtedly a brute force search method, and the efficiency of the algorithm itself cannot be ideal. The object-aware sampling method combined with the object pose information will eliminate the points that are impossible to generate grasp candidate from the point cloud based on the corresponding extracted features, that is, reduce the sampling space from the entire point cloud space to a specific space, cut down the number of invalid sampling and searching, improves the efficiency of the algorithm. According to the different acquisition of object pose information, the object-aware sampling method can be divided into three branches: object detection and segmentation, object affordance, and object shape complement. The three methods not only outperform object-agnostic sampling method in general scenarios, but can generate highly reliable pose candidates in their respective applicable environments. The pipelines of the relevant methods are provided in Figure 4.

##### Object Detection and Segmentation

The method based on object detection and segmentation is the earliest one of the three branches. This method first extracts the pose features of the object in the scene by taking the RGB images or the depth images of the scene or directly using the point cloud as input, and then segment the point cloud space to obtain a smaller and more reliable search space based on these features. Due to the introduction of the specific information of the object, this type of sampling algorithm has a significant improvement in the performance of the cluttered environment and collision detection. At the same time, with the rapid development of object detection and segmentation algorithms, YOLO (Redmon et al., 2016) and other efficient and easy-to-deploy backbone networks are widely used in this sampling method, and there is still a lot of work around its ideas.

By adopting sampling method in GPD proposed by Pas et al. (2017), Lopes et al. (2018), Schnaubelt et al. (2019), Bui et al. (2020), Chen et al. (2020), and Deng et al. (2020) sample the grasp points in point cloud for candidates generation. Lopes et al. (2018) find the largest planar surfaces which is infeasible for grasping by using RANSAC (Fischler and Bolles, 1981) and isolates the closest object to the camera from the rest of the scene to obtain object segmentation based on min-cut (Golovinskiy and Funkhouser, 2009). This work compares the experiments before and after reducing the point cloud search space, and proves that the grasping success rate has increased from 45 to 90%. Although the object's pose information is extracted with the help of object segmentation methods that are not based on deep learning, the impressive results show that the improvement is considerable. This shows that reducing the point cloud search space is a very reasonable and correct direction. Subsequently, more grasp candidate sampling methods integrated with object segmentation based on deep learning have been used in the development of this branch. Schnaubelt et al. (2019) segments the depth image by using Maskfusion (Runz et al., 2018) combined with increased noise robustness (Ückermann et al., 2012) and Bui et al. (2020) extracts object segmentation from point cloud with region of interest (ROI) obtained from YOLOv3 (Redmon and Farhadi, 2018). Deng et al. (2020) detects and segments the object from RGB-D images based on PoseCNN (Xiang et al., 2017), then a sampling method in Eppner et al. (2019) is adopted to generate 100 candidates for assessment and execution. Chen et al. (2020) utilizes object segmentation for mask-guide to improve the precision of sampling. Lin and Cong (2019), Lin et al. (2019), Sun and Lin (2020), and Yu S. et al. (2020) follow the same idea in GPD with additional physical or geometric constraints. Lin and Cong (2019) and Yu S. et al. (2020) adopt variant of PointNet (Qi et al., 2017a) and RANSAC for object segmentation respectively, then sample the grasp candidates with integrating physical and geometric constraints. In specific, the former work mainly considers the mechanical constraints in physics other than paying more attention to spatial constraints in the latter work. Lin et al. (2019) and Sun and Lin (2020) achieve object estimation via PPR-net (Dong et al., 2019) and Mask R-CNN (He et al., 2017) correspondingly followed by sampling the candidates based on using the closest ring of the centroid of the object. The main contribution and grasping accuracy are provided in Table 2.

**Table 2:** The summary of geometry-base object-aware grasp candidate generation.

**Method category**	**Work**	**Method backbone**	**Success rate (%)**	**End-effector**	**Environment**				**Simulation/reality**
					**Object arrangement**	**Object number**	**Object shape**	**Test novel object**	
Object detection and segmentation	Less is More (Lopes et al., 2018)	RANSAC	90	–	Single object	1	Irregular	No	R
	(Schnaubelt et al., 2019)	Maskfusion	–	–	Cluttered	5	Irregular	No	R
	RED (Chen et al., 2020)	Mask-RCNN + PointNet	84 (S) 82 (R)	Parallel-jaw gripper	Cluttered	7	Irregular	Yes	S/R
	(Bui et al., 2020)	YOLOv3	–	Parallel-jaw gripper	Single object	1	Regular	No	S/R
	(Deng et al., 2020)	PoseCNN	86.7	Parallel-jaw gripper	Cluttered	–	Irregular	Yes	R
	(Lin and Cong, 2019)	PointNet	90	Parallel-jaw gripper	Cluttered	5	Irregular	No	R
	(Yu S. et al., 2020)	RANSAC + VGG	–	Parallel-jaw gripper	Single object	1	Irregular	No	R
	(Lin et al., 2019)	PPR-net	78	Parallel-jaw gripper	Cluttered	30	Regular	No	R
	(Sun and Lin, 2020)	Mask R-CNN	71.1	Parallel-jaw gripper	Single object	–	Regular	No	R
Object affordance	(Qian et al., 2020)	ResNet101 + FPN	95	Parallel-jaw gripper	Single object	1	Regular	No	R
	TOG-Net (Fang K. et al., 2020)	SOM	80	Parallel-jaw gripper	Single object	1	Irregular	No	R
	kPAM (Manuelli et al., 2019)	Integral human pose regression	–	Parallel-jaw gripper	Single object	1	Regular	No	R
Object shape complement	(Varley et al., 2017)	CNN	93.33	Three fingers	Cluttered	–	Irregular	No	R
	(Lundell et al., 2019)	CNN	59	Parallel-jaw gripper	Cluttered	10	Irregular	No	R
	(Yan et al., 2019)	CNN	61	Parallel-jaw gripper	Cluttered	–	Irregular	Yes	R
	(Torii and Hashimoto, 2018)	DNN	85.6	Parallel-jaw gripper	Cluttered	–	Regular	No	S
	(Liu and Cao, 2020)	CNN	94.06	Parallel-jaw gripper	Cluttered	–	Irregular	Yes	R

##### Object Affordance

Affordance is introduced by Gibson (2014), which describes how likely the agent is capable to execute an action based on its surrounding environment. In robotics community, affordance, as a new physical and geometric property of objects, refers to the part of the objects with high probability to be operable. Specifically, affordance refers to the most likely part of the objects to make the grasping successful determined based on the knowledge of human grasping habits. Image that, people will always hold the handle of the hammer instead of the hammerhead when picking up a hammer, or hold the apple in the hand instead of grasping the apple stem when picking up an apple. Grasping pose sampling based on object affordance is an advanced method developed from object detection and segmentation methods. This method will determine the operable part with high possibility on the detected object. Since the search space in the point cloud is further reduced, the sampling results are more reasonable than that of grasp candidate sampling on the entire object. Concretely, if the target is a knife, if grasp sampling is performed on the entire object, those candidate grasps located on the blade will inevitably cause damage to the end effector of robots or other objects in the environment. Based on several methods to learn and understand object affordance proposed by prior art (Varadarajan and Vincze, 2012; Koppula et al., 2013; Katz et al., 2014; Zhu et al., 2014, 2015; Do et al., 2018), there has also been a lot of work in the grasp candidate generation based on the direction of object affordance.

Inspired by sampling integrated with traditional affordance detection methods (Pas and Platt, 2016; Kanoulas et al., 2017; Liu C. et al., 2019), diverse deep learning-based affordance-based sampling techniques are proposed. Qian et al. (2020) employs ResNet101 (He et al., 2017) with feature pyramid network (FPN) (Lin et al., 2017) to perform affordance detection and applies the sampling method proposed in Pas et al. (2017) with refined local reference frame computation. Instead, Fang K. et al. (2020) finds object affordance implicitly based on Mar et al. (2017) with a multi-dimensional continuous action space and uniformly samples grasps from the object surface using antipodality constraints (Mahler et al., 2017). Manuelli et al. (2019) detects keypoints of object affordance together with local dense geometric information instead of segmenting entire affordance, the reduced search space is able to guarantee a high-quality grasp candidates sampling (Gualtieri et al., 2016; Mahler et al., 2016, 2019).

Affordance learning is an advanced variant of basic segmentation method. Different forms of affordance such as semantic labels (Zhu et al., 2014), spatial maps (Jiang et al., 2012), and motion trajectories (Zhu et al., 2015) are suitable to diverse tasks, which is in a position to further reduce the search space and improve the confidence of candidates generation.

##### Object Shape Complement

Shape complementation is another improved variant of segmentation method. Unlike affordance learning attempts to understand the graspable components from detected objects, shape complement pays more efforts on “looking” the entire target object more completely. As another advanced branch developed from object detection and segmentation methods, shape complement and object affordance have completely different thoughts. Grasp candidate sampling based on object affordance is to reduce the sampling space to the area where the grasp is most likely to succeed, while shape completion is to try to minimize the occurrence of unreasonable sampling by obtaining more information about the shape of the object. In particular, for the point cloud captured by a monocular camera, it is impossible to outline the shape of the object where the light cannot reach. For symmetric objects, the effect of shape complement may not be so obvious, but for asymmetric objects, this type of methods is particularly conducive.

Varley et al. (2017) proposes a convolutional neural network which takes voxelized partial mesh of object as input and output the complemented shape. After a few post-preprocessing, GraspIt! (Miller and Allen, 2004) is used to generate grasp candidates. Lundell et al. (2019) improves the network architecture based on the method in Dai et al. (2017) by adding Monte-Carlo (MC)-Dropout (Gal and Ghahramani, 2016), an advanced dropout layer (Srivastava et al., 2014), into both training and run-time step to generate a set of shape samples. Grasp candidates sampling by GraspIt! is employed on the mean of shape samples. Yan et al. (2019) reconstructs object point cloud by integrating the segmentation via Mask R-CNN and several encoder-decoder modules (Fan et al., 2017) inspired by single-view 3D-object reconstruction (Jiang et al., 2018). Differ from complementing point cloud of object, Liu and Cao (2020) and Torii and Hashimoto (2018) leverage object primitives to simplify object shape under the detection output of convolutional neural network. Compared with GPD, their experiment results increased by 10.56 and 18%, respectively.

Although existing shape complement methods commonly accompany with high uncertainty, being aware of object shape is capable of incredibly facilitating the accuracy, robustness, and confidence of grasp proposals generation.

Object-aware methods aim to reduce the search space by being aware of the specific object, which improve the sampling performance. As shown in Table 2, the summary of geometry-based object-aware sampling method is listed.

### Learning-Based Sampling

**Figure 5:**
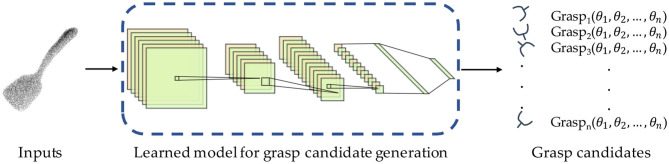
Learning-based sampling (The spatula point cloud is from YCB datasets).

Geometry-based sampling methods generate candidate grasp poses by changing the grasp configuration randomly under physical and geometric constraints in specific task, however, sampling a number of grasps poses proposals is computationally expensive. Furthermore, sampling the rotational or translational dimension possibly produce some unstable and unreasonable grasp configurations since the conditions in the grasp modeling are only artificially extracted. More recently, deep learning techniques improve performances in many traditional analytic tasks greatly based on more powerful feature extraction abilities compared to human handcraft. On the strength of deep learning, some researchers move from physical and geometric reasoning-based sampling to deep learning-based sampling as example in Figure 5. Different from the geometry-based sampling method, the learning-based sampling method will complete the learning based on a dataset during training. The learning-based methods can be divided into supervised learning or unsupervised learning according to the learning model. For sampling methods based on supervised learning, it takes scene point clouds or RGB images as input to obtain sampling results directly or extract appropriate grasp points first and then place the grasp posture on these points. It compares the prediction result with the ground truth to calculate the difference in sampling point determination, the difference in hand posture prediction. The sampling method based on unsupervised learning learns the distribution of sampling from the training data by the generative model, reducing the KL divergence or JS divergence to reduce the distribution difference between the data generated by the model and the training data. The trained model will be used for sample the grasp poses. Similar to geometry-based sampling, learning-based sampling methods fall into object-agnostic and object-aware group.

#### Object-Agnostic Sampling

Object-agnostic methods take the point cloud as input and generate the proposals by learned models without detecting the object in the point cloud. Compared with geometry-based object-agnostic sampling, although the learning-based object-agnostic sampling method also searches in the entire scene point cloud, it has the following improvements on the thoughts: (1) Geometry-based sampling determines whether the point is feasible to generate grasp pose only based on the spatial information in the point cloud (three coordinate values of x, y, z), while learning-based sampling can learn more high-level information through the models or networks to help improve accuracy. (2) the generalization of geometry-based sampling is not strong, especially for cluttered scenes, it often regards two very close objects as one, which leads to unreasonable results of grasp sampling. The learning-based sampling method is able to significantly improve this problem through the benefits of more extracted features. (3) Geometry-based sampling is essentially a simple random combination of various parameters. Although it has an advantages in amount, it also caused a sharp increase in the number of negative samples. In contrast, the sampling process of learning-based generation method is actually obtained by the prediction of the model, and the sampling result will contain more information about the effect of high-level features. With the development of deep learning, some researchers try in the direction of learning-based object-agnostic sampling methods using neural networks.

Jiang et al. (2020) proposes a deep convolutional neural network (DCNN) to predict the set of grasp points from the input depth-image. Inspired by Varley et al. (2015) that obtains grasps on pixels, Morrison et al. (2018a) presents a Generative Grasping CNN (GG-CNN) which generates candidates immediately on pixelwise. GG-CNN treats each pixel of image liberally without any hypothetical searching space, which may assure a higher probability of finding a global optimal grasp pose. Guan et al. (2019) adopt Fully Convolutional Neural Network (FCNN) (Long et al., 2015) to take four channels images synthesized by an RGB image and depth image as input and output three maps contain all information of potential grasp poses.

Theoretically, the learning-based object-agnostic sampling method should be better than the geometry-based grasp candidate generation method, but there is not much work in this direction. Moreover, in addition to some advantages in the efficiency of algorithm operation, there is no remarkable improvement in other aspects. This is mainly due to the limitations of this method: (1) The training dataset is difficult to generate. Since the grasping pose needs to be sampled by the prediction of the model, the ground truth label of the sample is difficult to represent. This results in the difficulty of model training. Although the careful design of the loss function and network structure can slightly improve this shortcoming, it may also cause a decrease in efficiency during training and testing. And some work is done by simplifying the objects in the dataset to complete the training, but this causes the generalization of the model to be very poor. (2) Although the model is used to complete the sampling process, due to the lack of specific information of the object, the essence of the algorithm is to perform a brute force search in the entire point cloud space. (3) The neural network-based model can indeed extract higher-level features, but because of the difficulty of ground truth representation and the lack of object specific information, the extracted features may not be too satisfactory, which leads to the performance of the algorithm is not outstanding.

#### Object-Aware Sampling

Geometry-based object-aware sampling first utilizes computer vision techniques to localize and segment object, then samples the candidates based on the reduced searching space. This method has shown the reliability and reasonability of generated candidates is improved observably, however, adopting handcraft constraints in sampling step may cause generating some unstable grasp poses and computationally expensive. To further address these issues, learning-based object-aware generation modes are proposed by researchers. These methods acquire several grasp candidates with the help of trained model after localizing and segmenting object.

Mousavian et al. (2019) and Murali et al. (2020) employ a sampler ground on variational autoencoders (VAE) (Kingma and Welling, 2013). The sampler's architecture is similar to GANs (Goodfellow et al., 2014), which takes in PointNet++ (Qi et al., 2017b) as encoder and decoder, aiming to generate several grasp candidates and determine how likely they are successful. Yu H. et al. (2019) doesn't localize and segment the object explicitly. The author presents regression network and refine network to regress an optimal grasp region, and sample and sort grasp candidates correspondingly. Zhao B. et al. (2020) uses two neural networks to segment the point cloud and generate grasp proposals correspondingly. Fang H.-S. et al. (2020) presents an end-to-end grasp pose prediction network given N point coordinates as input. Inspired by anchor-based progress in 2D object detection (Ren et al., 2015; Liu et al., 2016), Wu et al. (2020) adopts PointNet++ as backbone to build up a Grasp Proposal Network (GPNet) to generate a set of grasps. The generated proposals are pruned via two physical schemes which are removing grid corners not locate on the object surface and the contact points antipodal constraint (Chen and Burdick, 1993). Li Y. et al. (2020) proposes a Deep Residual U-Nets on the basis of residual modules (He et al., 2016) to predict the graspable region of object, which is followed by a K-means (Lloyd, 1982) model clusters the graspable point cloud and the center of each cluster is leveraged as a grasp point. Ardón et al. (2019) employs Markov logic networks (MLN) (Richardson and Domingos, 2006) for knowing the relationship between diverse objects and a pre-trained Res-Net (He et al., 2016) is utilized to accomplish object perception and feature extraction for querying grasp affordances by Gibbs sampling (Kim and Nelson, 1999). The main thought back Ardón et al. (2019) is sampling several grasp affordances and evaluate them, the affordance with highest possibility will be selected and corresponding grasp configuration is calculated. Inspired by leveraging rectangle represent grasp part (Jiang et al., 2011; Lenz et al., 2015), Vohra et al. (2019) and Yu Q. et al. (2020) sample numerous rectangles to characterize candidate graspable parts and gain the optimal grasp pose by filtering and scoring candidates. After catching sight of high efficiency and easy implementability of pixelwise sampling (Morrison et al., 2018a), Yu Y. et al. (2020) first detects the object via SSD (Liu et al., 2016), the detection results will be checked if the target object is occluded through clustered point cloud from K-means and an image inpainting and recognition network (IRNet) which is inspired by Yu et al. (2018) combined with light-weighted recognition network MobileNet (Howard et al., 2017). The detection and confirmation output are feed into a deep grasping guidance network (DgGNet) to generate and qualify the grasp in each pixel.

As shown in the table, the table compares the learning-based object-aware methods with GPD and GG-CNN, which are representative work of the geometry-based object-agnostic and learning-based object-agnostic sampling method. Although there is no universally applicable benchmark, based on the comparison of the success rate in the same grasp operating environment, it can be seen that the learning-based object-aware sampling methods have a significant improvement in the final grasp success rate in a cluttered environment. At the same time, the grasp success rate will be further improved for the single object.

Benefitting from the progress of several data-driven methodologies, learning-based object-aware models have the highest potential to fulfill grasp proposals selection. However, this type of method also has some drawbacks. One is that the sampling results obtained through prediction usually only consider the pre-shape of the end effector, which probably leads to unavoidable collisions during the motion planning process. The other is for multi-finger end effectors (excluding suction cups and parallel-jaw gripper), it is usually difficult for the model to predict the contact point of each finger, which will make the model perform satisfactorily in a cluttered environment tougher.

### Applicable Scene

Generally speaking, these grasp candidate generation methods can be tried on all tasks. But because the ideas of these methods are not the same, for some specific tasks, some methods will theoretically perform better than others.

#### Geometry Based or Learning Based?

Geometry-based grasp candidate generation is to extract the constraints in the mathematical modeling of the grasp to sample the possible feasible grasp poses in the point cloud. For scenes that are not very cluttered, this method is sufficient to sample a lot of reasonable grasp pose candidates, but if the operating environment is too cluttered, this type of method will be easy to wrongly judge two close objects as an object for sampling. Moreover, geometry-based grasp sampling requires additional conditions for collision detection. Although poor results may not necessarily occur, the efficiency of the algorithm will be greatly reduced.

The learning-based grasp candidate generation is based on a trained model, especially a neural network, which takes point cloud as input, and obtains the sampling result of the grasp pose according to the prediction of its output. Although this method is less interpretable and intuitive than the geomery-based method, the neural network is able to extract richer features in the hidden layer to help sampling, thereby reducing the computational difficulty of collision detection. However, the common problem of the learning-based sampling model is it often requires a lot of data to train a robust model. Collecting data and fabricate a dataset is expensive, which leads to the preparation process of the method time-consuming.

As shown in the Table 3, according to some experimental conditions, the recommendation of which grasp generation method to use is listed. This table only makes recommendations for the specific stage of grasp candidate generation. If combined with the evaluation stage, which is detailed in section Grasp Candidate Evaluation, there is no guarantee that geometry-based sampling will perform worse than learning-based sampling.

**Table 3:** Geometry-based and learning-based recommendation under different conditions.

**Condition**	**Recommendation reason**	**Recommendation**
Single object environment	Easy to sample grasp pose	Geometry-based
Collision-regardless	More constraints required to detect collision	
Hard to generate dataset	No training process based on large-scale dataset	
Cluttered environment	Sample grasp pose based on advanced features	Learning-based
collision-concern	No need to build hand-craft collision detection constraints	
easy to generate dataset	Suitable for training a model	

#### Object Agnostic or Object Aware?

As far as the current development in the field of robotic grasp is concerned, the object-agnostic sampling method is highly unrecommended. Object-agnostic sampling was proposed in the age when visual detection methods were not effective, but the lack of object information has a great influence on the generation of reliable grasp poses. Therefore, no matter what the task is, it is indispensable to add object information to the algorithm.

In the method of object-aware sampling, it can be subdivided into three branches: object detection and segmentation, object affordance, and shape complement. If it is only for a single object and its shape is regular, then the difference between these three methods will not be too obvious. But in reality, the operating environment of robots is not so ideal and simple. As the earliest developed branch, the method based on object detection and segmentation has a high degree of applicability. Regardless of the operation scenario, the pose information of the object can be extracted through the detection and segmentation of the grasped object, thereby helping the generation of grasping candidates. This kind of method is highly adaptable and can be used as a preliminary attempt in various tasks. Object affordance is to reduce the entire object in the sampling space to a more reliable local area. For simple regular objects, this type of method may not make much sense. However, this method is particularly important for objects that have a large deviation of the center of gravity or damage to the end effector of the robot. The shape complement method can be used as a solution to the poor quality of the input point cloud. The generation of the point cloud depends largely on the light conditions in the experimental scene. Sometimes the point cloud of some objects is very sparse due to poor lighting or the scene is too cluttered. In this case, direct sampling is not advisable. The shape complement is to restore the original shape of the object, so as to improve the information of the object and help the generation of the grasp candidate.

As shown in the Table 4, according to the experimental conditions, the recommendation of which grasp generation method to use is listed.

**Table 4:** Object-aware sampling branches recommendation.

**Condition**	**Recommendation reason**	**Recommendation**
Preliminary attempts	Highly adaptable	Object detection and segmentation
Regular object	Easy to detect and segment	
Harmless irregular object	Easy to detect and segment, no need to consider unsafe grasp pose	
Regular object	Able to detect more reasonable grasp	Object affordance
Irregular object	Able to detect where to grasp	
Harmful object	Able to detect a safe grasp	
Poor lighting condition	Restore object shape	Shape complement
Sparse point cloud	Restore object shape	
Irregular object	Filter unreasonable grasp pose with symmetric shape assumption	

## Grasp Candidate Evaluation

To execute the optimal grasp, a necessary step is to evaluate the generated grasp candidates previously. Evaluation is a latter portion aims to score grasp success probability or classify graspability of grasp proposals. By considering whether the approach is data-driven or not, evaluation methods can be divided into learning-free and learning-based. Learning-free determine each grasp a good or bad one based on geometry information or control system flow. On the other hand, learning-based attempts to acquire a model to perform as an evaluator trained from datasets.

### Learning-Free Candidate Evaluation

Learning-free candidate evaluation determine each grasp a good or bad one mainly based on geometry information. Since approaches in this group don't utilize learned models to carry out the assessment, they put efforts into converting proposals evaluation to an optimization problem. The thoughts behind this transformation is inspired by the optimal control theory in control system or reinforcement learning fields.

Zapata-Impata et al. (2017) presents a function to assess grasp configurations stability by considering the distance, direction and geometric shape of the grasp. On the contrary, Mahler et al. (2016) prefers to rank each grasp candidate based on physical conditions, especially force closure (Weisz and Allen, 2012; Kim et al., 2013; Laskey et al., 2015; Mahler et al., 2015). Following previous works solved the problem via Monte-Carlo integration (Kehoe et al., 2012; Weisz and Allen, 2012) or Multi-Armed Bandits (MAB) (Laskey et al., 2015), Mahler et al. (2016) takes in latter method to find the best grasp. Adopting the thoughts back of reinforcement learning, Manuelli et al. (2019) transforms the evaluation to an optimization problem which is used to find the desired robot action. The constraints of the optimization are established based on geometry, especially the distance of it.

Since learning-free approach normally perform the grasp selection under several assumptions and constraints which are simulated by geometry, causing the lack of flexibility and generality. There are only few works in mainstream take in this evaluation technique. In contrast, learning-based assessment has a wider variety.

### Learning-Based Candidate Evaluation

**Figure 6:**
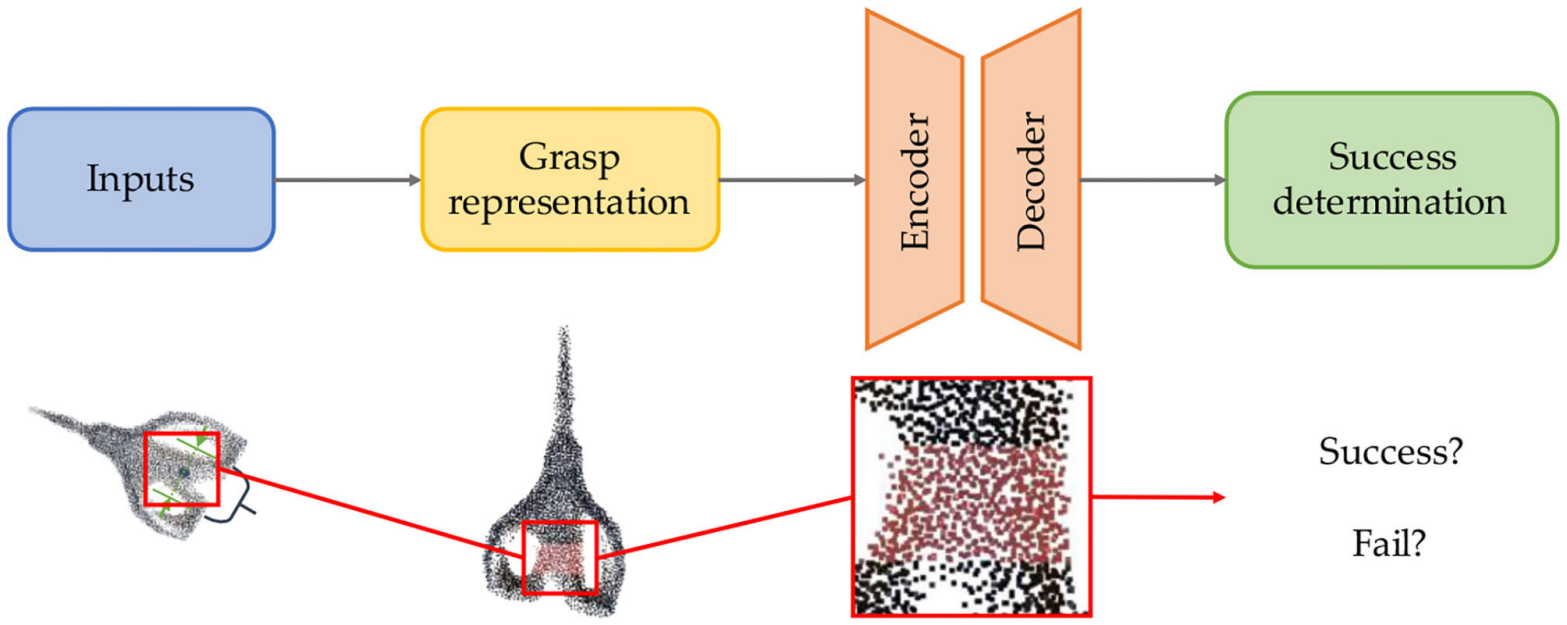
Learning-based candidate evaluation (The scissors point cloud is from YCB datasets).

Learning-based are widely used in evaluation step among numerous works. Due to assessing the grasp quality based on trained model from large datasets, learning-based methods are more robust and generalized than learning-free one with prior knowledge instead of complex analysis. Learning-based candidate evaluation pipeline example is provided in Figure 6. The learning-based grasp evaluation method is essentially a model that completes the binary classification task. The general pipeline at this stage is to first extract representation of grasp pose and grasp part, then use the learned model to finish evaluation. Whether it is based on SVM in the early days and later based on CNN, the essence of model training has not changed, which is to reduce the classification loss. From hinge loss to cross-entropy or other classification loss, the difficulty at this stage is not how to design the loss function, but how to effectively represent the grasp and use it as the input of the model for evaluation.

At early of the first, Le et al. (2010), Jiang et al. (2011), and Pas and Platt (2015) evaluate the grasp points by utilizing support vector machine (SVM) (Boser et al., 1992). SVM-based approaches are able to classify the suitable grasps with a good result in some simple cases or trivial problems. With the complexity growth of grasp scenarios, SVM, or other traditional data-driven techniques are decreasingly robust. To ensure the evaluation methods' capability of determining or scoring grasp candidates with noisy inputs, more researchers move their attention to deep learning-based evaluation methods.

Compared with traditional data-driven techniques, deep learning-based evaluation is more precise and robust. Kappler et al. (2016) first indicates the feasibility of evaluating based on CNN. Inspired by this, Gualtieri et al. (2016), Wang and Ling (2016), and Pas et al. (2017) use LeNet (LeCun et al., 1998) to classify the grasp proposals and achieve an impressive performance. Even LeNet architecture is shallow and are not robust to noisy grasp proposals, CNN-based classifier's potentials of evaluating grasps attracts large amounts of interests in robotic community. Following prior arts (Lenz et al., 2015; Gualtieri et al., 2016; Mahler et al., 2016), Mahler et al. (2017) and Mahler et al. (2018) propose a Grasp Quality Convolutional Neural Network (GQ-CNN) to evaluate grasp and suction task respectively. Depending on flexibility of modification, comprehensibility of architecture and simplicity of implementation, GQ-CNN becomes a wide preference among several works. Jaśkowski et al. (2018) utilizes a new CNN architecture and add batch normalization (Ioffe and Szegedy, 2015) to refine GQ-CNN. Mahler and Goldberg (2017) models bin picking on the basis of Partially Observable Markov Decision Process (POMDP) (Astrom, 1965) and fine-tunes GQ-CNN with a new dataset to evaluate actions instead of grasp configurations, which improves the generalization of GQ-CNN. Satish et al. (2019) further enhances GQ-CNN by designing a FC-GQ-CNN through fully convolutional network. Fang K. et al. (2020) proposes a Task-Oriented Grasping Network (TOG-Net) by making progress on GQ-CNN via residual network layers (He et al., 2016) to obtain task-agnostic grasp quality, conditioned task-oriented grasp quality and manipulation action. Although GQ-CNN achieve an impressive performance, it currently requests high-quality depth sensors to obtain desirable point cloud, which limits the deployment in many cases.

Except from GQ-CNN, other works also propose some novel evaluators to figure out grasp candidate assessment. Following 3D CNN predictor designed in Choi et al. (2018), Lou et al. (2020) passes voxelized point cloud of each grasp candidate into networks and fortify a reachability predictor to strengthen selected grasp robustness. Inspired by the work in Varley et al. (2015) and Lu et al. (2020) utilizes a patches-CNN to gain the information from different patches in images to calculate a suitable grasp. Van der Merwe et al. (2020) takes a further step by way of signed distance functions (SDF) to earn object reconstruction. The extracted point cloud embedding is set as the input to the success probability predictor (Lu et al., 2020) extended by collision-free strategy (Zhou and Hauser, 2017; Lu and Hermans, 2019; Lu et al., 2020). Qian et al. (2020) modifies fully connected layer by a novel pooling layer in R-FCN (Dai et al., 2016) which is able to perceive object localization change precisely. Yu Q. et al. (2020) classifies grasp rectangles via a 7-layer CNN. Although these variants of 2D CNN achieve impressive performances, a common drawback is it's hard for them to handle imperfect observed point cloud and extract sufficient and stable information.

Profiting from studies on point cloud (Rusu and Cousins, 2011; Guo Y. et al., 2020; Lu and Shi, 2020), PointNet and PointNet++ are two extraordinary and widespread models which make a further promotion on evaluation networks. Liang et al. (2019), Mousavian et al. (2019), and Yan et al. (2019) use PointNet to directly take point cloud as input and output the grasp candidate evaluation. The points within the closing area of the gripper are utilized to represent the grasp. Immediately transform grasp to the points in corresponding area addresses unstable prediction results from imperfect local observed point cloud and carries out an acceleration of evaluation process. Stimulated by PointNetGPD (Liang et al., 2019), Singh et al. (2018) tries to replace PointNet with PointNet++, KD-Networks (Klokov and Lempitsky, 2017) and Dynamic Graph Convolutional Neural Networks (DGCNN) (Wang Y. et al., 2019) to obtain a better result. By considering parallel structure utilized in some works ignores grasp candidate generation errors result in unreliable evaluation, Grasp Proposal Networks (GPNet) (Wu et al., 2020) adopts GraspNet (Mousavian et al., 2019) and designs a structure which is able to allow generator and evaluator to be trained jointly. Fang H.-S. et al. (2020) utilizes PointNet++ as their ApproachNet to obtain suitable grasps. In contrast with traditional 2D-input CNN, methods based on PointNet or PointNet++ are capable to handle noisy inputs and assess grasp candidate probability and stability with higher confidence.

Approaches presented by other arts are not in line with CNN-based methods but still achieve good performance. Enlightened by wide & deep model in recommender system (Cheng et al., 2016), Context-Aware Grasping Engine (CAGE) (Liu et al., 2020) treats discovering a feasible grasp configuration as a recommendation problem. Based on the prior knowledge, the model predicts suitable grasp by finding grasp configuration in the similar situation. On the contrary, Wu et al. (2019) utilizes a reinforcement learning pipeline, which assesses and refines the action taken in each time stamp based on reward function via policy gradient (Sutton et al., 1999).

## End-to-End and Others

Except from those works can be categorized into grasp generation or evaluation part, there still has numerous arts out of this framework. By adopting the advantages of end-to-end learning, some researchers attempt to concatenate two parts to enable training the network jointly. Instead of using supervised learning, some works empower grasping ability of robots through interacting with environments on the basis of reinforcement learning. Other proposed models are based on approaches not in mainstream.

### End-to-End Learning

With the development of deep learning, end-to-end learning gradually becomes one of the most outstanding learning mode, which is seemingly natural consequence of deep neural architectures blurring the classic boundaries between learning machine and other processing components by casting a possibly complex processing pipeline into the coherent and flexible modeling language of neural networks (Glasmachers, 2017). Deep learning based on end-to-end mode is capable of training and generating a more powerful model via a holistic object function.

S^4^G (Qin et al., 2020) proposes a single-shot grasp proposal network based on PointNet++ which assigns each point in point cloud a grasp configuration and its quality score. Non-maximum suppression (NMS) and weighted random sampling are applied to the output to select a grasp to be executed. Based on the network in (Choi et al., 2018), Liu M. et al. (2019) improves its performance through introducing a new combined loss which is composed of consistency loss and collision loss. These two losses aim to resolve grasp pose ambiguity and penalizes the penetrations respectively. Yu Y. et al. (2019) preprocesses the input via utilizing FPN with ResNet50 and K-means by taking RGB images and point cloud as inputs correspondingly to extract the multi-scale masks of target object. Then a DrGNet takes masks as inputs to perform depthwise separable convolution. The encoded results from DrGNet are refined by RefineNet (Nekrasov et al., 2018) and sSE (Roy et al., 2018) to obtain a desirable grasp. Provided by object mask, grayscale and depth images as input, Tosun et al. (2020) trains grasp proposal network (GPNet) (Tosun et al., 2019) and shape reconstruction network (SRNet) (Mitchell et al., 2019) parallelly to acquire grasp proposal and reconstructed point cloud. The embeddings from GPNet and SRNet are combined to refine the detected grasp. PointNetRGPE (Wang Z. et al., 2020) first predicts the corresponded class number from object point cloud data, which is used to fuse with point coordinates to pass into grasping pose estimation network. The network has three sub-networks based on PointNet to acquire the translation, rotation and rotation sign of grasp pose. Other than generate the grasp configuration parameters, GraspCNN (Xu et al., 2019) treats the grasp estimation as an object detection problem. It takes RGB images as input and outputs an oriented diameter circle. The circle and oriented diameter indicate the grasp area and gripper open width and closing orientation respectively. Obtained circle on RGB image is calculated to project into the point cloud.

Instead of designing end-to-end framework based on supervised learning, some arts propose their end-to-end models through the methodologies of reinforcement learning. Zeng et al. (2018a) attempts to jointly train two FCNs in Q-learning framework to extract the visual state representations and obtain the push and grasp from the policy. Compared with the demands of a large amounts of manually labeled data in supervised learning, approach presented in Zeng et al. (2018a) is totally self-supervised. Wu et al. (2019) takes depth images as input and obtain 10 2D maps after flowing in an FPN. Ten maps represent current state of end-effector and objects and are utilized to sample the action. Policy is learned based on policy gradient during the action execution. Manuelli et al. (2019) segments and detects the keypoints of object via Mask R-CNN and pose estimation network (Sun et al., 2018). The grasp planner (Gualtieri et al., 2016; Zeng et al., 2018b; Mahler et al., 2019) is selected to generate a pose and refined by proposed optimization method.

End-to-end models are capable of learning a complete function maps from visual inputs to grasp poses. Although there is not much work based on an end-to-end approach, this direction has great potential for development. Since it takes the point cloud as input, and then directly outputs the grasp pose, this omits the consideration of the features extracted from the connection in the multi-step method. At the same time, the efficiency of the end-to-end algorithm is usually better than that of the multi-stage model, which makes it possible for the robot to operate in real time in a dynamic environment.

### Reinforcement Learning

**Figure 7:**
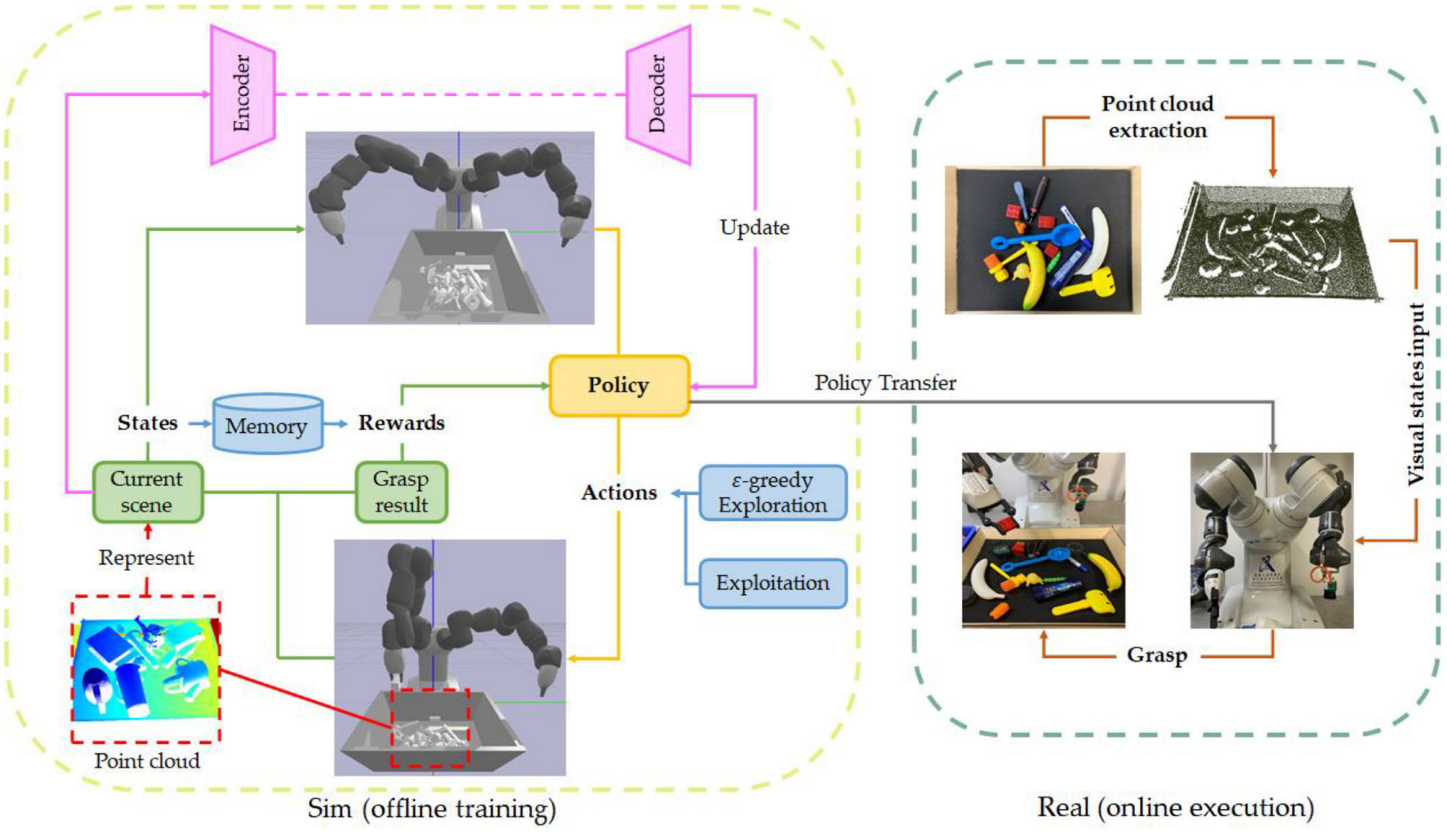
The deep reinforcement learning framework of robotic grasp learning based on point cloud. In order to reduce the cost of trial and error, the current robot grasping based on reinforcement learning is to first train the model in a simulation environment and then migrate to the real robot (Sim to real).

Reinforcement learning is another interesting solution of grasp pose detection. Although the work on reinforcement learning has been mentioned in the end-to-end method, what mentioned earlier is end-to-end learning based on the reinforcement learning framework. Generally speaking, reinforcement learning methods do not necessarily adopt end-to-end thinking, and vice versa. As shown in Figure 7, RL approaches enable robot to interact with environments to study the policy maps from visual inputs to actions. In contrast with supervised learning, trial and error thoughts back of RL capacitate robots to learn self-exploration ability, which makes robots have higher dexterity. The reason why reinforcement learning makes robots more dexterous than supervised learning is because the training ideas of the two are completely different. Supervised learning is to update the model parameters through sample and label pairs until the loss function is minimized. But the biggest drawback of this training method is that its dataset usually has only one label per sample. That is, for a grasp point, it can only correspond to one grasp pose, which actually adds a lot of restrictions to the model. Image that a robot wants to grasp a block or a ball, the same scene can have hundreds of thousands of grasp poses for humans because the deviation of the hand and joint angle has no effect on the successful grasp of such objects. However, there is only one correct answer in supervised dataset. On the contrary, reinforcement learning is to obtain the policy of grasping objects through trial and error. Since the robot may have explored many grasping possibilities during training, and the reward function value of each grasp pose is not bad, the algorithm will add these possibilities to the policy function, so that the possible answers for the operation become more.

Ficuciello et al. (2019) first take point cloud as input, an object recognition module is utilized to accomplish object detection and pose estimation. Acquired features are feed into a pretrained neural network to obtain the robot grasp initial configuration. Then a RL loop is used to refine the initialized parameters by assigning the executed grasp a cost to update the policy. Inspired by Deep Q-learning network (DQN) (Mnih et al., 2015), Gualtieri and Platt (2018) trains a CNN to learn Q-function and utilizes gradient Monte Carlo (Sutton and Barto, 2018) to update the rule. At each time stamp, it generate several grasp candidates based on hierarchical sampling and then one pose will be chosen by the learned policy. Chen et al. (2020) adopts RL to obtain an appropriate viewpoint based on the mask-guided award to perform GPD module. Rather than deploying RL strategy on grasp planner, this model focuses on acquire a better view sight to improve the grasp accuracy.

RL-based approaches empower robot ability of self-exploration from trial and error, successfully trained models are capable of planning grasping dexterously. However, methods based on reinforcement learning, especially when used for robot operations, will have a serious problem – the exploration space of the algorithm will become extremely large, or the grasp poses that require trial and error are innumerable. This will make the learning efficiency become very slow, and because the exploration space is too large, the positive and negative samples will be extremely imbalanced. The robot may not be able to increase the reward function value after many trial and error, and the policy cannot be updated. Besides, due to the necessity of a mass of sampling during training, the data collection process is also time-consuming. In addition, it still needs more time to transfer the model in simulation to real world since the cost of each trial may be extremely high.

### Others

Aside from end-to-end learning and reinforcement learning, some other works achieve the goal through some unique and creative methods.

Zhu et al. (2019) first obtains the graspable area via performing ellipse fitting method on segmented mask from Mask R-CNN. Then RANSAC is utilized to acquire the orientation of the grasp from the pixel mask and point cloud. Instead of generating the grasp pose parameters, Shao et al. (2020) attempts to predict the grasp contact points. It not only extracts the feature of object point cloud, but also pays attention to gripper properties. An unsupervised autoencoder adopts the structure of PointNet to learn a low-dimensional latent space of gripper representation and construct robotic hand representation from URDF file. Then the gripper representation and object point cloud features extracted by PointNet++ are combined to feed into a proposed Point Set Selection Network (PSSN) to generate correspond number of contact points based on beam search. Kokic et al. (2017) employs two CNNs, one for affordance detection and another one for classification and orientation estimation. Extracted parameters are used to compute a grasp by Haustein et al. (2017).

Approaches proposed in this group can achieve an impressive performance in some special cases, however, generalization of these methods are not approving.

## Learning Modes

There are numerous types of machine learning algorithms, which aim to solve problems in different situations or under diverse demands. In the robot grasp learning field, learning modes can be mainly divided into supervised learning, unsupervised learning and reinforcement learning even some arts may be outside of these three categories. Nowadays, due to the challenge of grasp pose detection with high dexterity, it facilitates the fusion of different learning modes to integrate the merits of each other to improve the model performance. The descriptions of three main modes are provided first, and then the fusion models are discussed based on three primitive modes.

### Supervised Learning

Supervised learning is the machine learning task of learning a function that maps an input to an output based on example input-output pairs (Russell and Norvig, 2002). The most prominent property of supervised learning is the datasets used to train the models have the labels for each sample. Supervised learning models are the most widely used since they are simple to implement, easy to train and suitable for most tasks. In robot grasp learning task, supervised learning methods can be used for candidate either generation or evaluation. Many end-to-end learning approaches are also based on the thoughts back of supervised learning.

Initially, supervised learning models without deep learning techniques are only used for grasp candidate evaluation since extensive demands of handcraft feature preprocessing cause low performance of candidate generation. Pas and Platt (2015) uses SVM to assess the quality of grasp proposals and acquires an incredible performance. With the development of deep learning, supervised learning models based on neural networks outperform and replace traditional techniques in many tasks. Gualtieri et al. (2016), Wang and Ling (2016), and Pas et al. (2017) try to evaluate the grasp pose candidates based on LeNet for the first time. Astonished by the power of neural network, deep learning models attract dramatically substantial interests from researchers. Mahler and Goldberg (2017) and Mahler et al. (2017, 2018) propose CNN-based evaluators with more complex architecture.

Inspired by several state-of-arts works in computer vision (Ren et al., 2015; He et al., 2016, 2017; Liu et al., 2016; Redmon et al., 2016), researchers begin to move the attention to grasp candidate generation. Some works attempt to use object detection and instance segmentation approaches to reduce the search space in the point cloud (Lopes et al., 2018; Schnaubelt et al., 2019; Bui et al., 2020) or regress the grasp part using rectangle bounding boxes directly (Vohra et al., 2019; Yu Q. et al., 2020). Specifically, models designed to process point cloud furtherly accelerate the progress of candidate generation (Lin and Cong, 2019; Yu S. et al., 2020) and evaluation (Singh et al., 2018; Mousavian et al., 2019; Yan et al., 2019; Fang H.-S. et al., 2020; Qian et al., 2020). Moreover, end-to-end learning models mentioned in section End-to-End Learning follow the supervised learning framework as well.

By adopting supervised learning methods, especially those in deep learning field, feature extraction of robot grasp learning has transformed from handcraft to learning-based. Supervised learning methods is capable of learning the models to accomplish specific tasks with only needs of regarding datasets. However, training an efficient supervised learning model requires a large amount of data. Data collection is very expensive and time-consuming in many cases which cause the model not able to learn enough knowledge to perform as expect, which is known as underfitting. In addition, incomplete training set also probably result in overfitting of models due to samples cannot represent the entire rules to be learned. For end-to-end learning, incorrect-design architecture has higher potentials to be overfitting. Besides, generalization of grasp learning is still a challenge. Model trained on one case is commonly hard to be transferred to other cases.

### Unsupervised Learning

In contrast with supervised learning, unsupervised learning takes unlabeled data as input aims to find the internal relationship of samples which allows for modeling of probability densities over inputs (Hinton et al., 1999). Two of the main techniques in unsupervised learning are dimensionality reduction and clustering. Clustering is used to group or segment the datasets to numerous clusters, which is adequate for processing the point cloud. Some works adopt K-means as clustering method to segment the point cloud as their desirable features for downstream.

Different from classical unsupervised learning approaches, VAE and GAN are the product of the growth of deep learning. Both VAE and GAN are generative models, which are to model the real data distribution from the training data, and then use the learned model and distribution to generate and model new data in turn. They are similar in two respects. One is that random noise is used in the data generation mode (such as Gaussian distribution is commonly used), and the other is that when modeling the distribution, it is necessary to measure the difference between the distribution of noise and training data. The difference between the two is essentially that the distribution measurement criteria are different (that is, the loss is different). VAE uses a more explicit measurement method, assuming that the training data is generated by another distribution, and directly measures the KL divergence of the training data and noise. From this, the ELBO theory, reparameterization trick and so on have been developed. The GAN cleverly avoids the direct measurement of the distribution difference, but lets the neural network learn this distance through confrontation. When the discriminator cannot distinguish between the two distributions, it is considered that the two distributions are consistent. The emergence of VAE and GAN makes neural networks also usable in tasks based on random sampling. Some works have adopted this strategy in the stage of grasp candidate generation. Inspired by GAN, Mousavian et al. (2019) and Murali et al. (2020) utilize VAE to sample multiple grasp proposals to speed up candidate generation.

Robot grasp learning is hard to be accomplished only based on unsupervised learning methods since study on unlabeled data is not trivial to acquire sufficient knowledge to drive robots perform dexterous grasp poses as human. However, unsupervised learning can efficiently segment point cloud or sample grasp candidates with satisfying results even using fairly simple models.

### Reinforcement Learning

Differs from demands of labeled and unlabeled data for supervised and unsupervised learning respectively, reinforcement learning (RL) aims to learn a policy maps from agent's states to actions and maximizes reward by interacting with environment. The core problem of RL is to find a balance between exploration and exploitation. The motivation of RL is letting the agent cumulate knowledge by trial and error. As mentioned in section Reinforcement Learning, there are few RL works achieve robot grasp goal based on point cloud and deep learning. The reason is the difficulty of transferring point cloud to visual state representation during robot exploration.

### Fusion

More recently, increasingly number of researchers begin to fuse different modes to adopt and integrate their advantages. Fused models enable employing the most appropriate learning types in each part of architecture, which allows a great promotion of grasp learning. It is very tough to model and accomplish a task only based on single learning modes. In addition, sometimes single mode perhaps complicates the problem and reduces the solving efficiency.

As mentioned in section Unsupervised Learning, it is hard to design a pipeline only based on unsupervised learning. K-means (Yu Y. et al., 2019, 2020; Li Y. et al., 2020) or GAN-analogous (Mousavian et al., 2019; Murali et al., 2020) methods only serves one functional part in entire architecture. On the other hand, like the discussion in section Reinforcement Learning, it is significant to acquire valid state representation to obtain a powerful reinforcement learning model. By integrating the visual perception, it is intuitive to utilize the input RGB images or point cloud to extract state information. Object localization and segmentation are almost achieved by numerous supervised learning-based CNN. Therefore, reinforcement learning techniques are naturally fused with supervised learning. However, large-scale datasets are commonly demanded in supervised learning to obtain a high-performance model, which request time-consuming data collection and annotation.

Self-supervised learning is a new mode in machine learning. Instead of paying expensive cost of gathering and labeling data, as a subset of unsupervised learning, self-supervised learning aims to study the labels represented by features based on provided unlabeled data. In robot learning, it's pretty costly to collect datasets and annotate each sample manually. In addition, defining, labeling and representing ground-truth for each sample may be challenging and wrong annotation can cause the model hard to train. Self-supervised learning is able to extract the feature-based labels during studying to avoid inaccuracy of handcraft. Zeng et al. (2018a), Deng et al. (2020) and Fang K. et al. (2020) adopt self-supervised framework to learn the model with a few data to pretrain or without any data to train from scratch. The results are surprisingly good and exhibit the great potential to employ self-supervised learning into robot learning research. Moreover, since training process is on the basis of unannotated data, the model performance can become cumulatively better with growth of learning time. Self-supervision is looked forward to promoting the continual learning of robotics.

## Applications

**Figure 8:**
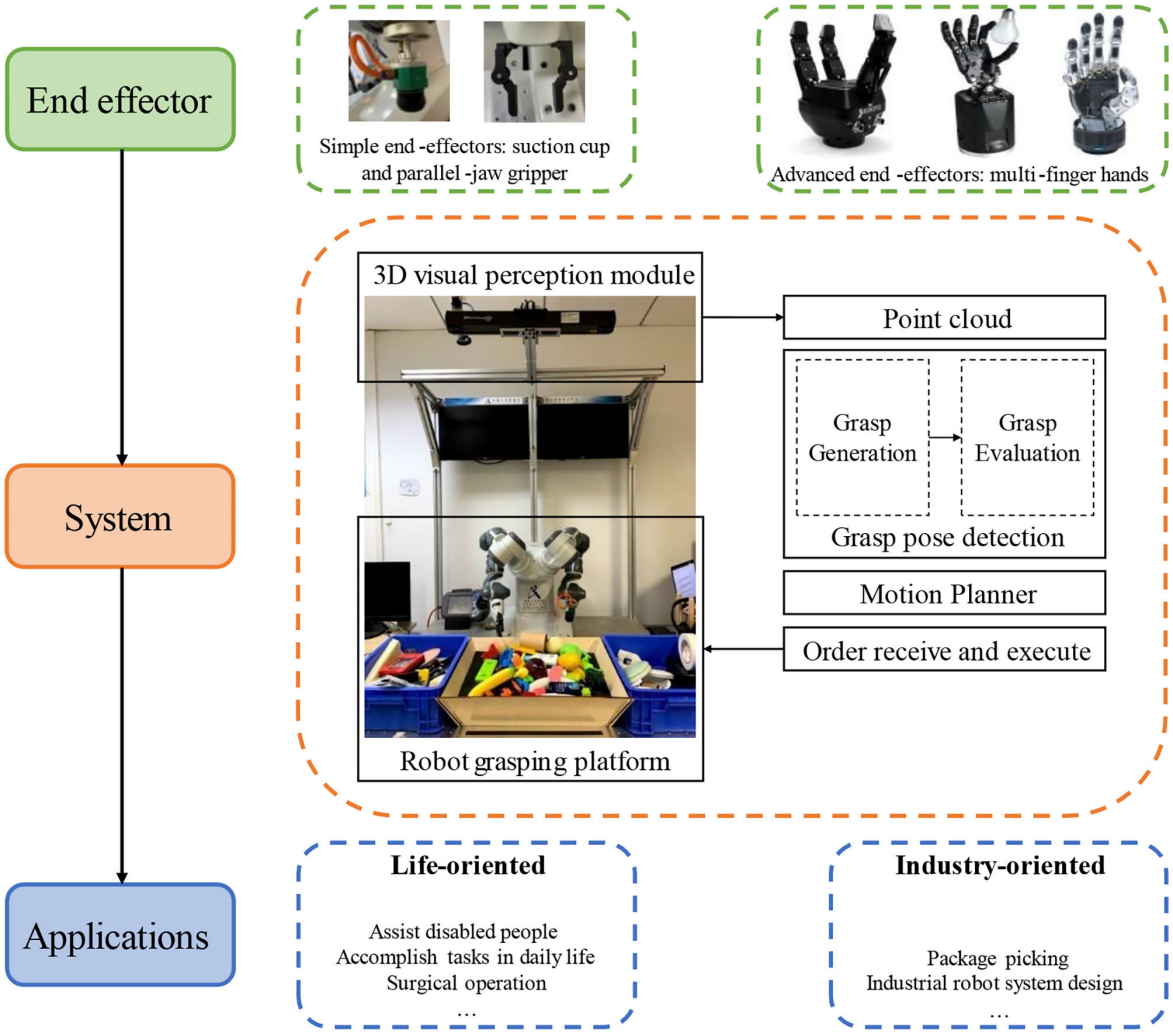
End effector, system and applications for robotics dexterous grasping. End effector is divided into simple end-effectors and advanced end-effectors. The former group contains suction cup and parallel-jaw gripper, the latter class indicates those multi-finger hands. Grasping system is first designed, deployed, and matured on simple end-effectors, then transferred and improved on advanced end-effectors. Developed systems are applied in different scenarios, life-oriented, or industry-oriented.

There are numerous applications of robot grasping based on point cloud and deep learning. Generally, as shown in Figure 8, these methods can be categorized from two different concepts. From the perspective of end effector, approaches can be divided into suction cup, two fingers (parallel-jaw gripper) and multi fingers. In terms of operating scenarios, approaches fall into life-oriented and industry-oriented.

### End Effector

Due to grasping mechanisms and DOFs vary from single suction cup (one finger) to five-finger hand, end effector plays an important role in grasp learning algorithm design. With more fingers assembled on end effector, the dexterity of grasp increases dramatically to allow robot to accomplish more complicated tasks.

#### Suction Cup

The suction manipulation based on vacuum cup has an unparalleled advantage over other multi-finger operation, which just request detect one feasible contact point to perform object picking. Single contact operation is not only convenient, and there is no need to worry too much about the collision between the end effector and other objects, especially in cluttered environment. Jiang et al. (2020), Mahler et al. (2018), and Mahler et al. (2019) pay efforts to pick the object through suction and obtain 98, 95, and 97.5% success rate respectively.

#### Parallel-Jaw Gripper

Robots equipped with parallel-jaw gripper are more favored in research and real life than humans grasp an object using movable joints index finger and thumb. Compared with suction manipulation, parallel-jaw gripper is able to perform more dexterous operations even if it demands more consideration of collision. The main reasons are grippers are easy to model in physical simulation environment and sample the grasp poses in point cloud. By knowing the grasp configuration and location in operating space, it is straightforward to calculate the contact points via geometrically symmetric shape to detect the collision. Pas and Platt (2015), Gualtieri et al. (2016), Mahler et al. (2017), Pas et al. (2017), and Liang et al. (2019) are fairly representative arts in the field of two-finger grasping.

#### Multi Fingers

There are merely few works focus on multi-finger grasp learning, not only is it difficult to model in the simulation environment, but also because of its overhigh DOFs and contact points make the sampling-based candidate generation strategy more sophisticated. Despite this, there are still some works focus on multi-finger grasping have achieved satisfactory results. Guan et al. (2019), Lin and Cong (2019), Liu C. et al. (2019), Wu et al. (2019), Shao et al. (2020), and Yu Y. et al. (2019), Yu Y. et al. (2020) utilize three-finger hand as their end effectors and Ficuciello et al. (2019) and Yu Q. et al. (2020) adopt five-finger to design grasp learning algorithm.

### Operating Scenarios

To categorize approaches in terms of operating scenarios, they can generally be divided into life-oriented and industry-oriented groups. Life-oriented methods aim to design a robot system can serve or help people to accomplish tasks in their daily life. Industry-oriented methods are designed to complete those dangerous and arduous tasks in industry.

#### Life-Oriented

Grasp is one of the most primitive and core manipulations of robot, many advanced operations are variant of grasping. With the growth of robot grasp learning, more and more tasks can be finished by robot in people's daily life, which improves quality of people's life. Gualtieri et al. (2017) and Zhang and Demiris (2020) propose robot systems to assist disabled people grab objects and dress cloths. Llopart et al. (2017), Zhou et al. (2019), Yang et al. (2020) and Zeng et al. (2020) aim to learn grasping capability to accomplish opening doors, grabbing glasses, picking objects from human's hands and throwing arbitrary objects. More interestingly, Parhar et al. (2018), Guo N. et al. (2020), and Kang et al. (2020) utilizes robot grasp ability to help completing crops harvesting in the farm. Zhang et al. (2020) enables robot grasping to be controlled by a smartphone. Hu et al. (2019) creatively integrates robotic grasp with surgery, which may help doctors during surgical operation. Nishikawa et al. (2019) and Schnaubelt et al. (2019) let the robot learn the knowledge to aid rescuers clean up disaster scene.

#### Industry-Oriented

Some works are aimed at industrial scenarios and propose algorithms for robot grasping learning to further promote productivity. Tian et al. (2017), Antonova et al. (2018), Li et al. (2018), Li H. et al. (2020), Song et al. (2019), Bui et al. (2020), and Liu et al. (2020) present some system design idea based on robot grasping that can be used in industrial production. Amazon Picking Challenge (APC) is a competition to provide a challenge problem to robotics community (Wurman and Romano, 2015) that spawns numerous excellent works based on the combination of point cloud and deep learning. Hernandez et al. (2016), Zeng et al. (2017), Morrison et al. (2018b), and Matsumoto et al. (2020) are some typical picking systems designed in top teams.

## Challenges and Future Directions

Robot grasp learning based on 3D point cloud provides more potentials to estimate and execute more precise grasp pose on the target objects. Deep learning growth also brings more promising strategies on grasping system design. Applications mentioned in section Applications exhibit the great probability of dexterous-grasping ability can be closely integrated with tasks in a variety of industries. However, due to sparse and unstructured properties of point cloud, training difficulty and low generalization of deep learning and ambiguity of dexterous grasping definition, the challenges of robot grasp learning are provided. Some future directions are also discussed based on these unresolved problems. Challenges and directions can mainly be divided into three groups, improving perception and manipulation abilities of robots, promoting the intelligence of robots and enabling abilities of transferring.

### Easy-Vision and Complicated-Dexterity

To achieve successful grasp goal, proposed methods heretofore commonly utilize multi viewpoints input and simplified grasp definition. Suppose deploying floor mopping robots, it is difficult to install a camera in each room. Instead, the camera will be equipped on robot which is able to barely provide partial observation of many objects. Hence, easy vision becomes increasingly useful. “Easy” means robots only take partial observation of objects from single viewpoint to detect the grasp pose. Concerning the grasp definition, lots of works are on ground of multiple hypothesizes and posture restrictions, which are not the true sense of 6-DOF grasping. Moreover, as mentioned in section End Effector, most arts focus on grasp learning with parallel-jaw gripper, which cannot be said as genuine dexterous grasping.

#### Single-View Grasping

Considering training a formidable grasping models, numerous works capacitate robot's visual perception ability by offering visual training data collected from multi-viewpoints. In spite of this can achieve a high grasping accuracy in special cases, these approaches have an important assumption which is robot has a complete knowledge of the environments to perform grasp poses. As a result of impossibility and hardness of providing entire object observation in diverse task scenarios, single-view grasp learning plays an increasingly important role. Shape complementation (Watkins-Valls et al., 2019; Van der Merwe et al., 2020) or taking partial observation as input immediately (Yan et al., 2019; Qian et al., 2020; Qin et al., 2020) are tried by researchers. Even sophisticating the model design and training, single-view grasp learning enhances algorithm robustness and reduces data collection cost.

#### 6-DOF Grasping

To accomplish grasping task in a simple way, many works stipulate the end effector can only grasp objects along the axis perpendicular to workspace. However, these restrictions make performed grasps lose the so-called 6-DOF. In addition, these analogous 6-DOF grasps are hard to achieve a satisfying accuracy in cluttered environment. 6-DOF grasping should be flexible that is capable of approaching the detected grasp point from any directions. With the progress of grasping learning models and robot hardware, the problem of quasi-6-DOF grasping has been gradually improved (Gualtieri and Platt, 2018; Lin and Cong, 2019; Mousavian et al., 2019; Lou et al., 2020; Murali et al., 2020).

#### Multi-Finger Grasping

The ultimate goal of robot grasp learning is to give the robot an anthropomorphic grasping ability, and it is a particularly important step to evolve the end effector from parallel-jaw gripper to five fingers. Thanks to the simple characteristics of modeling and physical analysis of parallel-jaw gripper, many works have achieved incredible results on the problem of two-finger grasping. However, as for multi-finger robots, especially five-finger robots, effective work is still lacking (Ficuciello et al., 2019; Yu Q. et al., 2020). High hands DOF and computational complexity caused by multi-contact points and multi-joints remain to the difficult enhancement of five-finger grasping.

### Fusion, Self-Exploration, and Continual Learning

Promoting the intelligence of robots is an appealing and core field in the future directions. Learning algorithms give robot intelligence, and the quality of the algorithms is the most prominent dependent factor for accurately and dexterously grasping objects. In order to design more complementary algorithms, fusion has become the current general trend. Fusion not only includes the integration of multiple learning modes, but also the integration between multiple modalities. Furthermore, enabling robots to have the ability of self-exploration and continual learning with a few prior knowledges has also become a hot Research Topic.

#### Fusion

As mentioned in section Fusion, the fusion of learning modes adopts the most appropriate learning modes at each part of the method architecture to improve model performance and reduce learning costs. In the contrast with multi-mode fusion, multimodal fusion not only relies on point cloud itself to improve the ability of grasp learning, it also utilizes language or tactile sense to enrich the features extracted in the learning process so that the robot has more grasping knowledge (Sung et al., 2017; Zhou Y. et al., 2018; Abi-Farraj et al., 2019; Kumar et al., 2019; Ottenhaus et al., 2019; Wang T. et al., 2019; Watkins-Valls et al., 2019).

#### Self-Exploration

Self-exploration refers to the method in which the robot learns to grasp through interacting with environments. Self-exploration breaks away from traditional approaches of relying on supervision but turns the problem into learning a policy maps from states to actions via trial and error. Reinforcement learning is currently the most powerful tool for self-exploration. As mentioned in section Reinforcement Learning, some works have been carried out around RL and achieved impressive results. Nevertheless, because the reward function is difficult to design, the deep reinforcement learning training requires a large-scale dataset support and the generalization performance is poor, there is still a lot of room for development of robot self-exploration based on RL.

#### Continual Learning

Continual learning (CL) is a machine learning paradigm where the data distribution and learning objective change through time, or where all the training data and objective criteria are never available at once (Hadsell et al., 2020; Lesort et al., 2020). Even for a grasping task, it is currently tough to grasp different types of objects in different scenes by a single robot. Continual learning allows the robot to learn new manipulation knowledge while not forgetting what has learned before. This ability makes it possible for a robot to complete multiple tasks. However, due to the demands for a large amount of memory to store the learned knowledge, and to adapt to the changes in the data distribution at any time, there is not much work in related fields as present. Moreover, continual learning and self-exploration complement each other. As the number of trial and error increases, robots will learn more and more knowledge. How to ensure that new knowledge doesn't overlap, conflict and cover the previous knowledge will require continual learning strategy.

### Sim-to-Real and Generalization

Designing, training, and deploying a grasp learning model is often time-consuming and laborious. Researchers expect to apply transfer learning strategies (Pan and Yang, 2009) to the learned model, so that similar grasping tasks don't need to be restarted from scratch. At present, there are three crucial problems in robot learning have not been well-solved. First is the problem of sample efficiency. Because the data for training robots is difficult to collect and a lot of collected data will not be helpful to the promotion of model learning due to unrepresentative. The other is the generalization ability of the model. Since the robot's end effectors are diverse and grasp learning is for specific tasks, the generalization ability is mostly poor. Finally, sim-to-real is also a challenge. Unlike traditional artificial intelligence tasks, robot learning commonly involves training and testing algorithms in a physical simulation environment and then transplanting to real robots. However, because the simulation environments are based on the ideal physical situation, it is quite different from the reality. Moreover, the migration of visual algorithms obtained in the simulation environments to reality will have cross-domain problems, which will cause the performance of the algorithms drop significantly after migration.

#### Sample Efficiency

In robot learning, especially those methods based on reinforcement learning, sample efficiency is particularly significant due to the particularity of the data. The low sample efficiency will not only increase the cost of data collecting and model training, but also cause the model to easily underfit. For the off-policy reinforcement learning approaches, since the policy is improved based on training samples, models with high sample efficiency can quickly learn appropriate policy from the data (Gualtieri and Platt, 2018; Zeng et al., 2018a; Zeng, 2019).

#### Model Generalization and Transfer

The methods mentioned in sections Grasping Candidate Generation and Grasp Candidate Evaluation based on CNN to generate and evaluate grasp candidates show the potentials of generalization (Mahler et al., 2017, 2018; Yan et al., 2019; Chen et al., 2020; Shao et al., 2020). However, for the proposed approaches based on reinforcement learning are hard to be transferred to other tasks. Even if there are already some works on the integration of transfer learning and reinforcement learning (Tirinzoni et al., 2018; Ammanabrolu and Riedl, 2019; Gamrian and Goldberg, 2019; Liu Y. et al., 2019; Xu and Topcu, 2019), the work related to the robot grasping is lacking. The essence of transfer learning is the registration problem at the task level, including not only the task itself, but also its input and output. If robot grasping can be defined from a higher level, if will make transfer learning possible.

#### Sim-to-Real

Simulation-based training provides data at low-cost, but involves inherent mismatches with real-world settings (Zhao W. et al., 2020). At present, domain randomization and domain adaptation are widely used in sim-to-real problems. In order to not degrading the performance, these methods attempt to make the data distribution from the simulation environments and real-world environments more similar to each other. Nonetheless, the low interpretability of domain randomization approaches and non-real hypothesis of domain adaption still make the sim-to-real hard to solve. Imitation learning, meta-learning and knowledge distillation are also supposed to have probability to facilitate the solution, however, more time is requested to achieve the goals.

## Conclusion

The current researches on robot dexterous grasp learning based on point cloud and deep learning can be divided into grasp candidate generation and grasp candidate evaluation. On the basis of this effective and reliable two-stage algorithm model, this survey proposes a more generalized learning framework. Most of the work can be summarized as a substantial contribution to one of these two stages. For work that does not belong to this framework, aside from the end-to-end model, the most prominent part is reinforcement learning framework. Although reinforcement learning is not summarized in the main framework of this review, sampling grasping actions from policy and assessing grasping actions from reward function can actually be regarded as the idea of proposed framework. But in order to respect the original motivation of the authors, RL-based approaches are not categorized. This survey aims to provide valuable insights and inspiration ground of sufficient bibliographical contents. Although there are still numerous challenges and limitations, methods with point cloud and deep learning have proven their potentials in promoting the improvement of robot dexterous grasping.

## Author Contributions

HD and PW brought up the core concept and architecture of this manuscript. HD and YH collected the majority of references. HD wrote the paper. GX and WW processed the objects point clouds of figures in the paper. XS refines some details of expression in section Object-Aware Sampling. All authors contributed to the article and approved the submitted version.

## Conflict of Interest

The authors declare that the research was conducted in the absence of any commercial or financial relationships that could be construed as a potential conflict of interest.
